# Effects of polyphenol-rich seed foods on lipid and inflammatory markers in patients with coronary heart disease: a systematic review

**DOI:** 10.3389/fnut.2024.1493410

**Published:** 2024-11-19

**Authors:** Yatian Jia, Hui Wang, Wen Fan, Jie Lv, Qingmei Niu, Ruifang Zhu, Qian Zhang

**Affiliations:** ^1^Nursing Department, Shanxi Bethune Hospital, Shanxi Academy of Medical Sciences, Third Hospital of Shanxi Medical University, Tongji Shanxi Hospital, Taiyuan, China; ^2^School of Nursing, Shanxi University of Chinese Medicine, Yuci, China; ^3^Editorial Office, First Hospital of Shanxi Medical University, Taiyuan, China

**Keywords:** polyphenols, coronary heart disease, blood lipids, inflammatory markers, systematic review

## Abstract

**Background:**

Coronary heart disease (CHD) is a prevalent cardiovascular condition, with its incidence and mortality rates steadily rising over time, posing a significant threat to human health. Studies have indicated that polyphenols exhibit a certain degree of protective effect against coronary heart disease. However, the findings regarding the impact of polyphenol-rich seed foods on patients with CHD have yielded inconsistent results.

**Objective:**

This study investigated the effects of polyphenol-rich seed foods on blood lipids and inflammatory markers in patients with coronary heart disease.

**Methods:**

The China National Knowledge Network, China Science and Technology Journal Database, China Biomedical Literature Database, Wanfang Database, PubMed, Cochrane Library, Embase, and Web of Science were searched for articles from the self-built database until March 16, 2024. The quality of the included studies was assessed using Edition 2 of the Cochrane Randomized Trials Risk Bias Tool, and data analysis was conducted using RevMan 5.4.

**Results:**

The study encompassed seven articles, with a total participation of 324 patients diagnosed with coronary heart disease. The study incorporated three seed foods abundant in polyphenols: Brazil nut, almond, and flaxseed. The meta-analysis findings revealed a significant reduction in triglyceride levels [MD = −20.03, 95% CI (−32.25, −17.44), *p <* 0.00001] among patients diagnosed with coronary heart disease who incorporated seed-based foods abundant in polyphenols into their diet regimen. Furthermore, a notable enhancement was observed in HDL cholesterol levels [MD = 3.14, 95% CI (1.55, 4.72), *p* = 0.0001]. Moreover, the type of intervention substance influenced the observed effects. The consumption of almonds has been demonstrated to significantly reduce total cholesterol [MD = −15.53, 95% CI (−21.97, −9.1), *p* < 0.00001] and LDL cholesterol [MD = −14.62, 95% CI (−20.92, −8.33), *p* < 0.00001] in patients diagnosed with coronary heart disease. Additionally, the incorporation of flaxseed into the diet has shown an enhanced effect on reducing C-reactive protein levels.

**Conclusion:**

The consumption of polyphenol-rich seed foods can moderately improve TG and HDL-C levels in patients with coronary heart disease, while incorporating flaxseed into their diet can effectively improve inflammatory markers.

## Introduction

The prevalence and mortality of coronary atherosclerotic cardiopathy (CHD), commonly known as coronary heart disease, are progressively increasing year by year due to evolving lifestyle patterns, posing a significant threat to human health and life. The global health economy has been significantly burdened by this condition, which currently stands as the leading cause of mortality in the United States. It is estimated that 10.9% of adults aged 45 and above, along with 17.0% of adults aged 65 and above, are afflicted by coronary heart disease in the United States (1). According to the fifth China Health Service Survey in 2013, the prevalence rate of coronary heart disease among individuals aged 60 and above in China stands at 27.8‰, with a concomitant upward trend observed in mortality rates over time (2). The findings of several studies suggest that dyslipidemia and inflammation play a pivotal role in the pathogenesis of coronary heart disease (CHD) (3, 4). Currently, lifestyle modifications, pharmacotherapy, and revascularization procedures are the primary treatment modalities for CHD (5). Antiplatelet therapy serves as the cornerstone for secondary prevention of cardiovascular diseases; however, a subset of patients exhibit intolerance toward secondary prevention therapy, with up to 52.1% of individuals suffering from CHD displaying resistance to aspirin (6). Additionally, there exists a potential risk of bleeding (7). After revascularization, patients are required to adhere to a regimen of various medications, including antiplatelet and lipid-lowering agents, while still potentially encountering complications such as restenosis and postoperative depressive symptoms (8, 9). The occurrence of cardiovascular diseases is closely associated with diet and lifestyle (10), and the significance of “diet and lifestyle” as the fundamental aspect in preventing cardiovascular disease risk has been acknowledged by members of the European Atherosclerosis Society. Therefore, diet adjustment is essential for preventing and treating coronary heart disease. There is an urgent necessity to identify a safe and cost-effective dietary regimen for the management and treatment of coronary heart disease.

Polyphenols are a class of bioactive compounds widely distributed in plants, which contribute to the maintenance of health and exert preventive, delaying, or reducing effects on the occurrence and progression of certain chronic diseases. They are considered beneficial in combating degenerative conditions like atherosclerosis and central nervous system disorders (11), while also playing a preventive and therapeutic role in various medicinal and food homologous substances with favorable anti-inflammatory, antioxidant, and lipid-lowering properties (12). The impact of diet on cardiovascular disease development has been well-documented (13). Consequently, there is a growing interest among food and medical researchers in polyphenolic diets, necessitating the exploration of dietary plans that are not only safe and convenient but also highly compliant. Polyphenolic compounds have been found to be abundant in a diverse range of seed-based foods (14). Therefore, this study conducted a systematic review and meta-analysis of randomized controlled trials investigating the effects of polyphenol-rich seed foods on patients with coronary heart disease. The aim was to determine the efficacy of these foods in regulating blood lipids and inflammatory markers, providing valuable insights for future dietary management programs aimed at preventing cardiovascular diseases.

### Methods

The present study has been duly registered with PROSPERO (registration number: CRD42024532025)^1^ and adheres to the guidelines outlined in the Preferred Reporting Items for Systematic Reviews and Meta-Analyses (PRISMA) statement (15) within this paper.

The following inclusion and exclusion criteria were formulated to investigate the potential impact of seed foods rich in polyphenols on blood lipid levels and inflammatory status in patients with coronary heart disease.

### Inclusion criteria

1. Patients aged ≥18 years diagnosed with coronary heart disease based on angiography or myocardial; 2. Patients provided with a dietary intervention involving any of the 22 seed foods listed in the Phenol Explorer Food Polyphenol Content Database^2^ with no limitation to the grams, quantity, and duration of intervention seed food; 3 Control group for comparison with participants administered either the standard diet or a placebo.

### Exclusion criteria

1. Patients who had additional comorbidities; 2. Patients where intervention incorporated additional active compounds or medications; 3. Patients taking seed food extracts; 4. Repeated published studies; 5. Articles such as research proposals, conference papers and abstracts that lack or be devoid of access to primary data.

### Search strategy

The search was conducted independently by two researchers who systematically searched eight databases, including CNKI, VIP, Wanfang Data, China Biomedical Literature Database (CBM), PubMed, Cochrane Library, Embase, and Web of Science. The search period spanned from the inception of the database until March 16, 2024. Please refer to Annex 1 for the detailed search strategy.

### Literature screening and data extraction

Two researchers independently conducted a comprehensive literature search, importing the retrieved articles into EndNote20 literature management software for further analysis. Subsequently, the titles and abstracts were carefully reviewed to exclude irrelevant studies, followed by a thorough examination of the full texts to select relevant articles based on predefined inclusion and exclusion criteria. The extraction of data from selected studies was performed independently by both researchers, encompassing information such as the first author’s name, publication year, participants involved, sample size, intervention measures employed, intervention duration, outcome indicators assessed, among others. In case of any discrepancies during this process, consultation with a third researcher was sought.

### Literature quality assessment

The included literature was assessed by two investigators using the Cochrane Randomized Trial Bias Risk Assessment Tool, Edition 2 (RoB 2) (16) evaluation criteria. The evaluation encompassed the following aspects: randomization process, deviation from expected interventions, missing outcome data, outcome measures, and selection of reported outcomes. Based on the results obtained from The RoB 2 assessment tool, each article was categorized as “high risk,” “some concern,” or “low risk.” In case of discrepancies during the above process, a third researcher would act as an arbitrator to facilitate consensus-building.

### Evidence quality assessment

The certainty of the body of evidence was assessed using the Grading of Recommendations, Assessment, Development and Evaluations (GRADE) approach. The quality of evidence for RCTs was initially rated as high in this methodology, but it would be downgraded to medium, low, or very low if any limitations related to bias, inconsistency, directness, imprecision, or risk of publication bias were identified. The evidence, however, can be enhanced by incorporating significant effects and dose–response gradients. In the event of disagreement during the aforementioned process, a third researcher will serve as an arbitrator to ultimately reach a consensus.

### Data analysis methods

The meta-analysis was conducted using RevMan 5.4 software. The mean square error (MD) combined effect size was utilized for the collection of quantitative data, and each point estimate of the effect size along with its corresponding 95% confidence interval (CI) was computed. According to the guidelines of the Cochrane Manual, I^2^ values were utilized for assessing inter-study heterogeneity, with *I*^2^ values ranging from 0 to 40%, 30 to 60%, 50 to 90%, and 75 to 100%, indicating no significant, low, medium, and high levels of heterogeneity, respectively (17). A random effects model was utilized, and further subgroup analysis was conducted based on potential sources of heterogeneity. Sensitivity analysis was employed to assess the stability and precision of the findings. A narrative analysis was performed for outcomes that exhibited excessive heterogeneity.

## Results

### Literature search results

**Figure 1:**
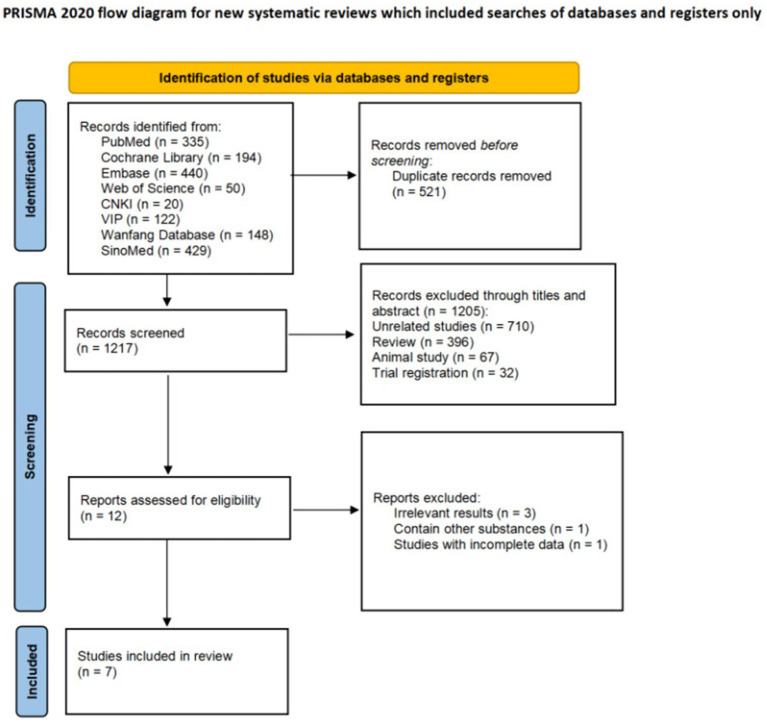
Literature screening process.

After conducting an initial search of the database, a total of 1738 relevant literature sources were retrieved. Subsequently, by eliminating 521 duplicate sources, the final count amounted to 1,217 literature sources. After reviewing the title and abstract, a total of 1,205 literature sources that clearly did not meet the inclusion criteria were excluded, while 12 literature sources that potentially fulfilled the inclusion criteria were identified. The final selection comprised a total of seven relevant studies (18–24). The process and outcomes of the literature screening are illustrated in Figure 1.

### Basic characteristics of the included studies

A total of seven articles (18–24) were included in this study, among which six were randomized controlled trials (18, 20–24), and one was a randomized cross-controlled trial (19). The study included two articles (22, 23) with 324 patients from Iran, Brazil, the United States, and Pakistan. Among the seed foods examined were Brazil nuts, almonds, and flaxseed. Notably, almonds were identified as a medicinal and food homologous substance. The duration of the intervention varied between 6 weeks and 3 months (Table 1).

**Table 1:** Basic characteristics of the included literature.

First author, publication, year	Country	Study design	Patients	Age year	Sample size(T/C)	Sex (male/ female) (T/C)	Intervention, time	Control condition	Dietary guidance	Outcomes	Time points of measurements	Outcome details
Cardozo (18)	Brazil	RCT	patient with CHD	T: 63.3 ± 6.7C: 63.3 ± 8.0	42(25/17)	T:13/12C:8/9	5 g Brazil nut a day, 3 months	Refrain from consuming any other types of nuts and maintain a daily nut-free diet, 3 months	Refrain from consuming any other types of nuts and adhere to the original dietary plan.	① a,b,c,d	after 3 months	The levels of TC, LDL-C, HDL-C, and TG did not show any significant changes.
Chen et al. (19)	America	Cross RCT	patient with CHD	61.8 ± 8.6	45	27/18	The National Cholesterol Education Program (NCEP) Step 1 Diet was supplemented with a daily intake of almonds (85 g/day), excluding other types of nuts, 6 weeks	The National Cholesterol Education Program (NCEP) Step 1 Diet, 6 weeks	Reinforce the implementation of the NCEP Step 1 diet, emphasizing a dietary pattern with reduced saturated fat and cholesterol intake.	① a,b,c,d ② CRP	after 6 weeks	The almond diet did not yield any significant impact on plasma lipid profiles or CRP levels.
Coutinho-Wolino et al. (20)	Brazil	RCT	patient with CHD	T: 62.7 ± 6.8C: 63.7 ± 8.7	37(23/14)	T: 11/12C: 6/8	One Brazil nut a day, 3 months	Refrain from consuming any other types of nuts and maintain a daily nut-free diet, 3 months	Refrain from consuming any other types of nuts and adhere to the original dietary plan.	① a,b,c,d ② CRP	after 3 months	The supplementation of Brazil nuts for a duration of 3 months did not result in any significant changes in TC, LDL-C, HDL-C, or TG levels in patients with CAD.
Jamshed et al. (21)	Pakistan	RCT	patient with CHD	32–86	113 T_1_:T_2_:C = (34/38/41)	113/37 (baseline)	T1: Consumption of 10 g/day Pakistani almonds, 12 weeks. T2: Consumption of 10 g/day American almonds, 12 weeks. (Soak the almonds overnight and consume them after peeling, before breakfast).	Refrain from consuming almonds and other nuts in your daily diet, 12 weeks	Refrain from consuming any other types of nuts and adhere to the original dietary plan.	① a,b,c,d	after 6、12 weeks	The consumption of almonds leads to a significant increase in HDL-C levels.
Khandouzi et al. (22)	Iran	RCT	patient with CHD	T: 56.67 ± 7.44C: 56.22 ± 9.02	44(21/23)	T:16/5C:21/2	Take a daily dosage of 30 g of flaxseed, 12 weeks	Receiving standard care, 12 weeks	Aim to consume a minimum of five servings of fruits and vegetables per day, while opting for foods that have reduced levels of saturated fat and cholesterol.	① a,b,c,d	after 12 weeks	The addition of flaxseed to the diet of patients with CHD did not result in any significant impact on plasma lipids.
Khandouzi et al. (23)	Iran	RCT	patient with CHD	T: 56.67 ± 7.44C: 56.22 ± 9.02	44(21/23)	T:16/5C:21/2	Take a daily dosage of 30 g of flaxseed, 12 weeks	Receiving standard care, 12 weeks	Aim to consume a minimum of five servings of fruits and vegetables per day, while opting for foods that have reduced levels of saturated fat and cholesterol.	② CRP	after 12 weeks	The inclusion of flaxseed in the diet of patients with CHD leads to an improvement in plasma inflammatory markers.
Saleh-Ghadimi et al. (24)	Iran	RCT	patient with CHD	T: 55.67 ± 6.9C: 54.8 ± 7.80	40(21/19)	T: 19/2C: 17/2	200 mL of sterilized milk with 1.5% fat content, supplemented with 2.5% flaxseed oil, 10 weeks	200 mL of sterilized milk with 1.5% fat content, 10 weeks	The participants adhered to a moderately calorie-restricted dietary regimen throughout the study.	① a,b,c,d	after 10 weeks	The consumption of flaxseed oil has been shown to effectively decrease triglyceride levels in patients with coronary heart disease (CHD).

① Blood lipid:a. LDL-C; b. TC; c. HDL-C; d.TG; ② Inflammatory biomarker: C-reactive protein (CRP); RCT: randomized controlled trial; T: treatment group; C: control group.

### Methodological quality of studies

**Figure 2:**
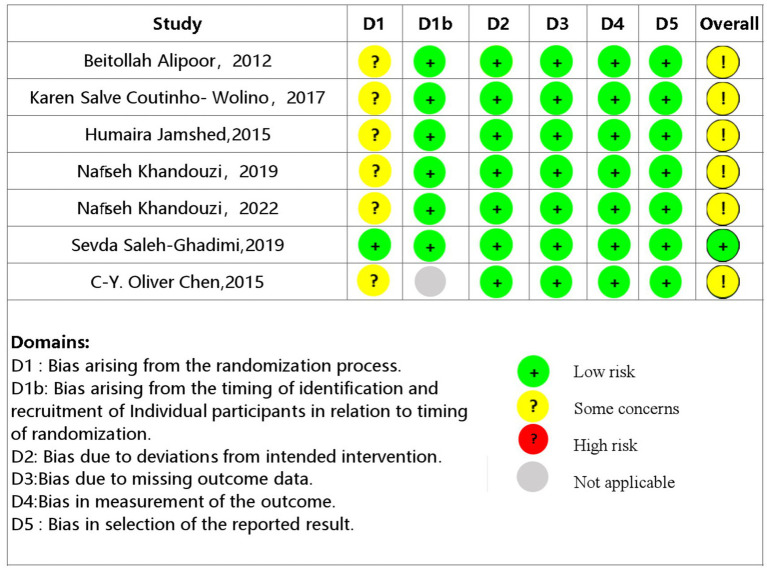
Results of methodological quality evaluation.

The two researchers independently utilized the Cochrane Bias Risk Assessment tool (RoB 2) to evaluate the methodological quality of the included studies. Although all the included studies reported randomized controlled trials, there was evidence of bias in the randomization process, with only one study (24) providing detailed information on the randomization method and assignment concealment. The results of the assessment on the methodological quality of the included literature are presented in Figure 2.

### Quality of evidence

According to the GRADE manual, TG and HDL-C have been assigned a low quality rating for evidence, while TC and LDL-C have been given a very low quality rating. The evaluation details are in Table 2.

**Table 2:** Quality assessment.

Quality assessment							Effect	Quality	Importance
No of studies	Design	Risk of bias	Inconsistency	Indirectness	Imprecision	Other considerations	Rate (95% CI)		
TC									
6	Randomized trials	Serious^1^	Serious^2^	No serious indirectness	Serious^3^	None	MD: −4.19 (−15.25 to 6.87)	ÅOOO VERY LOW	CRITICAL
TG									
6	Randomized trials	Serious^1^	no serious inconsistency	No serious indirectness	Serious^3^	None	MD: −20.03 (−32.25 to −17.44)	ÅÅOO LOW	CRITICAL
LDL-C									
6	Randomized trials	Serious^1^	Serious^2^	No serious indirectness	Serious^3^	None	MD: −2.00 (−11.31 to 7.3)	ÅOOO VERY LOW	CRITICAL
HDL-C									
6	Randomized trials	Serious^1^	No serious inconsistency	No serious indirectness	Serious^3^	None	MD: 3.14 (1.55 to 4.72)	ÅÅOO LOW	CRITICAL

HDL-C: high-density lipoprotein cholesterol; LDL-C: low-density lipoprotein cholesterol; TC: total cholesterol; TG: triglyceride; CHD: coronary heart disease; CRP: C-reactive protein.

### Effect of polyphenol-rich seed foods on TC in patients with coronary heart disease

**Figure 3:**
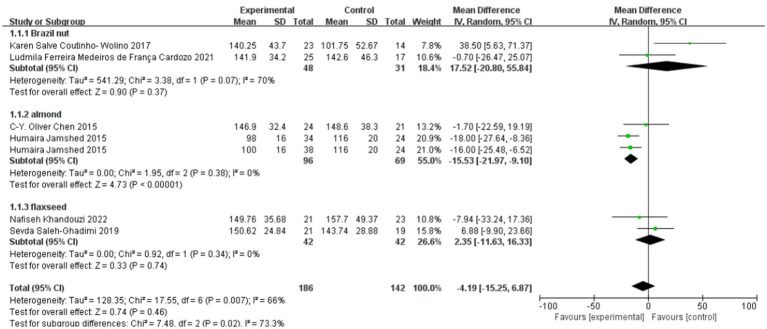
Effect of polyphenol-rich seed foods on TC in patients with CHD.

The effect of polyphenol-rich seed foods on total cholesterol (TC) in patients with coronary heart disease was investigated in six studies (18–22, 24). One study (21) included two intervention groups: Pakistani almond and American almond. The heterogeneity test results revealed a significant level of heterogeneity, with *p* = 0.007 and *I*^2^ = 66%. Subgroup analysis based on intervention seed foods demonstrated that the almond group exhibited a significant reduction in TC among patients with CHD [MD = −15.53, 95%CI (−21.97, −9.1), *p* < 0.00001]. However, the impact of Brazil nut and flaxseed groups on total cholesterol (TC) in patients with coronary heart disease (CHD) did not yield statistically significant results. The overall findings from the meta-analysis indicate that the consumption of polyphenol-rich seed foods does not have a significant effect on TC levels in individuals with CHD [mean difference = −4.19, 95% confidence interval (−15.25, 6.87), *p* = 0.46]. See Figure 3.

### Effect of polyphenol-rich seed foods on TG in patients with coronary heart disease

**Figure 4:**
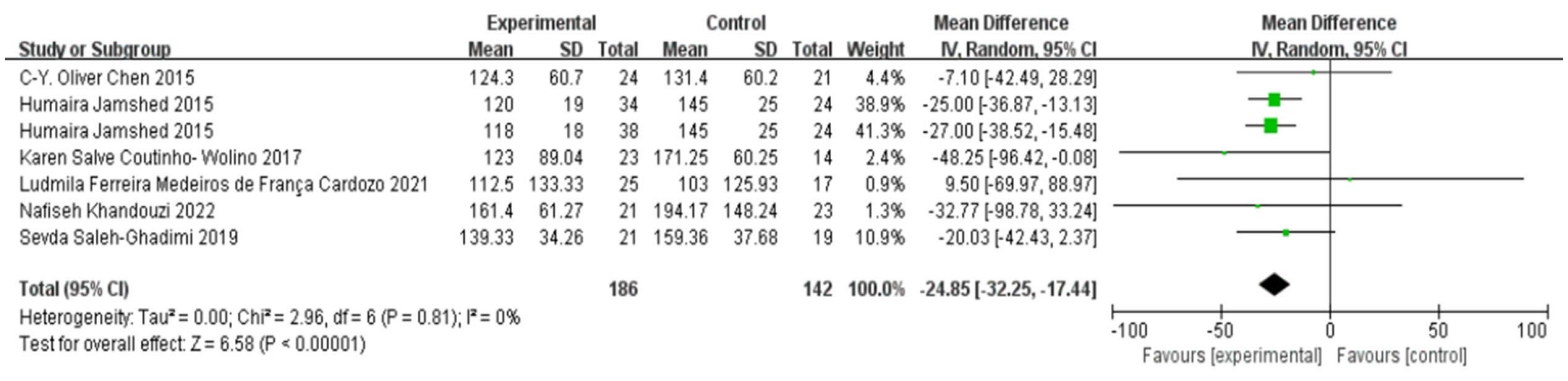
Effect of polyphenol-rich seed foods on TG in patients with CHD.

The effect of polyphenol-rich seed foods on TG in patients with coronary heart disease was investigated in six studies (18–22, 24). Heterogeneity test results demonstrated no significant heterogeneity (*p* = 0.81, *I*^2^ = 0%). Meta-analysis results revealed a statistically significant reduction in TG levels among patients with coronary heart disease who consumed polyphenol-rich seed foods [MD = −20.03, 95% CI (−32.25, −17.44), *p* < 0.00001]. See Figure 4.

### Effect of polyphenol-rich seed foods on LDL-C in patients with coronary heart disease

**Figure 5:**
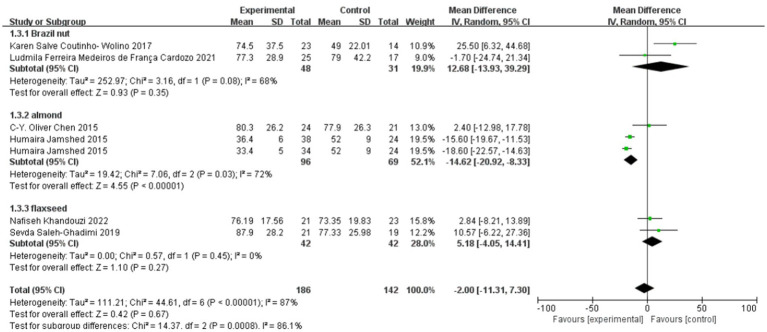
Effect of polyphenol-rich seed foods on LDL-C in patients with CHD.

The effect of polyphenol-rich seed foods on LDL-C in patients with coronary heart disease was investigated in six studies (18–22, 24). Heterogeneity test results revealed significant heterogeneity (*p* < 0.00001, I^2^ = 87%). Subgroup analysis based on intervention seed foods demonstrated a significant reduction in LDL-C among patients with coronary heart disease who consumed almonds [MD = −14.62, 95% CI (−20.92, −8.33), *p* < 0.00001]. However, no significant effects were observed for the Brazil nut and flaxseed groups regarding CHD patients’ LDL-C levels. Overall meta-analysis results indicated that the consumption of polyphenol-rich seed foods did not significantly affect LDL-C levels in patients with coronary heart disease [MD = −2.00, 95% CI (−11.31, 7.3), *p* = 0.67]. See Figure 5.

### Effect of polyphenol-rich seed foods on HDL-C in patients with coronary heart disease

**Figure 6:**
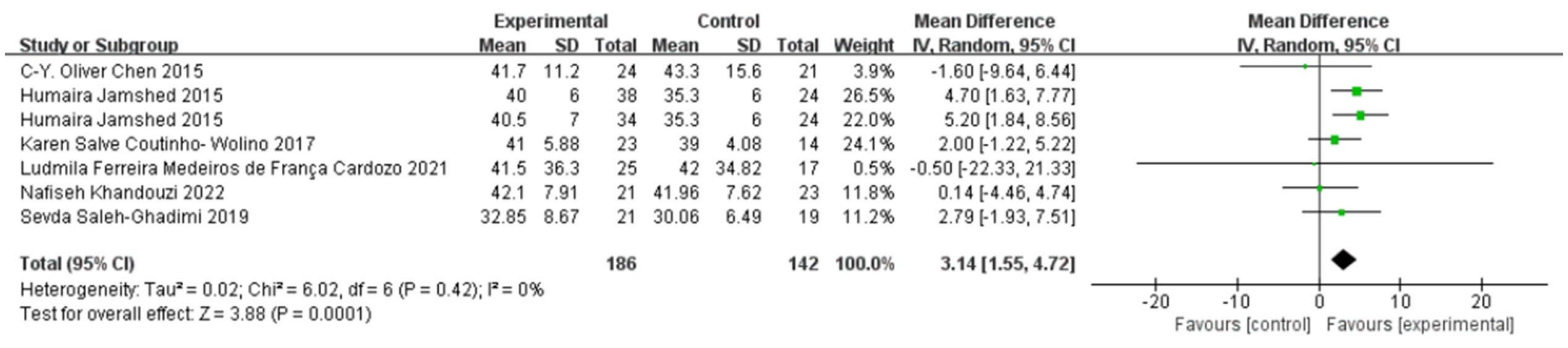
Effect of polyphenol-rich seed foods on HDL-C in patients with CHD.

The effect of polyphenol-rich seed foods on HDL-C in patients with coronary heart disease was investigated in six studies (18–22, 24). Heterogeneity test results indicated no significant heterogeneity (*p* = 0.42, *I*^2^ = 0%). Meta-analysis revealed a significant positive impact of polyphenol-rich seed foods on HDL-C levels in patients with coronary heart disease [MD = 3.14, 95%CI (1.55, 4.72), *p* = 0.0001]. See Figure 6.

### Effect of polyphenol-rich seed foods on CRP in patients with coronary heart disease

Three studies (19, 20, 23) examined the impact of polyphenol-rich seed foods on CRP levels in patients diagnosed with coronary heart disease. Among these studies, one (23) demonstrated a significant improvement in CRP levels through the incorporation of flaxseed into the diet, while no significant improvements were observed with almonds and Brazil nuts (19, 20).

### Sensitivity analysis

The sensitivity analysis revealed no significant alterations in the combined findings, and no individual study was found to exert a substantial influence on the statistical outcomes, thereby indicating the relative stability of the meta-analysis results.

## Discussion

### Effects of polyphenol-rich seed foods on blood lipids and inflammatory markers in patients with coronary heart disease

In this study, it was observed that consumption of polyphenol-rich seed foods significantly improved the levels of triglycerides (TG) and high-density lipoprotein cholesterol (HDL-C) in patients diagnosed with coronary heart disease. The seed foods investigated in this study included almonds, Brazil nuts, and flaxseed. Notably, almond consumption demonstrated a significant reduction in total cholesterol (TC) and low-density lipoprotein cholesterol (LDL-C), while incorporating flaxseed into the diet resulted in decreased levels of the inflammatory marker C-reactive protein (CRP).

Almonds, among the foods examined in this study, are recognized as medicinal and food homologous substances according to traditional Chinese medicine theory. They possess dual properties of being both food and medicine, playing a beneficial role in the prevention and management of various chronic diseases. The concept of “homologous medicine and food” aligns with the principles of natural, green, and healthy living, as well as resonates with the traditional Chinese medicine philosophy of disease prevention through holistic approaches (25, 26). In the process of foraging, human beings have systematically documented the inherent qualities and therapeutic potential of various types of sustenance, gradually recognizing that numerous edibles possess medicinal properties, blurring the distinction between food and medicine. The Qianjin Prescriptions, a traditional Chinese medicine text, states: “If one can utilize food to alleviate diseases and promote recovery, they can be deemed as an adept practitioner.” Apart from providing essential nutrients for the human body, food also exerts influence on the equilibrium and regulation of human physiological functions. With prolonged adherence to this process, its effects become increasingly evident (27).

The medicinal and food homologous substances encompass a diverse range of polyphenols, particularly flavonoids, which serve as efficacious constituents in the reduction of blood lipids (28). Meanwhile, polyphenols in drug and food analogs have been scientifically proven to possess anti-inflammatory properties, primarily by inhibiting the metabolic pathway of prostaglandins (29). The lipid levels are closely associated with the development of coronary heart disease. An intervention study conducted on patients with hyperlipidemia demonstrated that incorporating almonds into their diet not only reduced lipid risk factors for coronary heart disease, but also significantly altered the relationship between these risk factors and susceptibility to lipid oxidation modification (30). A meta-analysis on almond consumption revealed significant improvements in blood lipid levels and inflammatory markers (31, 32), which aligns with the findings of this study. The study suggests that polyphenols, such as flavonoids, positively influence the cardiovascular system through their antioxidant and anti-inflammatory properties. These effects may enhance endothelial function through various molecular mechanisms, regulate vasodilation processes, and modulate nitric oxide (NO) levels by inhibiting NAD(P)H oxidase (33).

The findings of nutritional epidemiological studies often demonstrate a robust association between increased phenol intake and a decreased risk or incidence of non-communicable diseases (34). The studies have revealed a shared characteristic among chronic non-communicable diseases, namely the presence of low-grade, persistent, and systemic inflammation. Furthermore, oxidative stress and lipid abnormalities are also recognized as significant contributors to the development of chronic illnesses (35, 36). The previous research conducted by this research team (12) has concluded that various healthy diet patterns share commonalities and posits that non-nutrients present in food have the potential to promote health, prevent diseases, and aid in the treatment of chronic diseases through their anti-inflammatory, antioxidant, and metabolic regulatory properties. The research team simultaneously introduced a “theoretical model of family nurse diet therapy,” in which oxidative stress, inflammation, and metabolic disorders in chronic illnesses form an equilateral triangle with non-nutrients at its apex, creating a triangular pyramid structure. Nurses employ dietary prescriptions to prevent and manage chronic conditions while incorporating fundamental principles of wholesome eating patterns derived from both traditional Chinese medicine and contemporary medical perspectives (12). A cross-sectional study investigating the association between total nut consumption (including both tree nuts and peanuts) and metabolic health revealed a significant inverse relationship between higher nut intake and the incidence of hypertension, type 2 diabetes, and dyslipidemia. Moreover, an increased consumption of almonds was specifically associated with a reduced risk of developing hypertension. These findings suggest that incorporating nuts into one’s diet may benefit metabolic status and prevent chronic diseases (37). The findings of a study demonstrated a correlation between the consumption of beverages rich in polyphenols and the presence of cardiovascular and metabolic risk factors. The consumption of polyphenol-rich beverages has been observed to be associated with higher intake of polyphenols, thereby reducing the risk of cardiovascular disease. It is worth noting that the Mediterranean diet places emphasis on consuming foods abundant in polyphenols, such as fruits, vegetables, whole grains, etc. (38). The study conducted by Zhang et al. revealed that the bioactive compounds present in medicinal and food homologous substances can effectively support the treatment of non-alcoholic fatty liver disease (39). A meta-analysis of lipids and apolipoproteins in nuts indicated that flavonoids may exert anti-inflammatory effects by attenuating LDL oxidation and modulating the expression of inflammatory genes in endothelial cells and macrophages (40).

The consumption of natural foods rich in polyphenols has been found to be safe for human consumption. Two studies (19, 21) included in the analysis reported no adverse reactions or safety concerns among patients. In the traditional Chinese diet, homologous substances of medicine and food are utilized as daily food ingredients, serving a specific role in health preservation. However, improper consumption of these substances may result in potential toxic side effects that can harm human health. The European Commission Regulation (EC) stipulates a maximum daily intake of 1,000 mg of polyphenol extract consumed by humans (41). The field of traditional Chinese medicine also acknowledges the interplay and potential contradictions between medicinal and food homologous substances (42). The content of non-nutrients in food intake should be carefully considered when supplementing non-nutrients in daily life, ensuring the appropriateness of food consumption.

Some of the included studies observed a lack of positive effects on patients, which may be attributed to variations in intervention timing, polyphenol content, and food preparation methods. The duration of intervention in the included studies varied from 6 to 12 weeks. Humaira Jamshed et al. demonstrated a significant increase in HDL-C levels among patients with coronary heart disease by incorporating almonds into their diet for a period of 12 weeks (21). Conversely, another study focusing on almonds observed no significant impact on the plasma lipid profile of patients after a 6-week intervention (19). In two studies investigating the impact of polyphenol-rich apples on patients with hyperlipidemia (43, 44), Athanasios Koutsos’ study (43) utilized fresh apples containing polyphenols for a duration of 8 weeks, with an apple weight of 340 g. This intervention resulted in a significant improvement in blood lipid levels among individuals with hyperlipidemia. Conversely, one study (44) examined the effects of freeze-dried apples containing polyphenols over 4 weeks, equivalent to approximately 270 grams of fresh apples. However, no notable effect on blood lipids was observed among patients with hyperlipidemia. The study on the impact of almond consumption on adult inflammatory biomarkers revealed that when the daily intake of almonds was less than 60 g, it reduced serum CRP levels (31). Therefore, the duration and dosage of intervention may influence the efficacy of polyphenols in patients. Additionally, the food preparation method can also impact both the polyphenol content in food and its effects on individuals with coronary heart disease. Two studies on flaxseed have shown that dietary addition of flaxseed does not significantly affect plasma lipids, while consumption of flaxseed oil can effectively reduce triglycerides (TG) (22, 24). A study on cereal polyphenols (45) also reported that different processing methods of cereal impacted its polyphenol content. Therefore, in the process of managing diets for patients with coronary heart disease, it is recommended to encourage the consumption of seed foods as part of a healthy diet to help maintain healthy blood lipid levels, reduce inflammation levels and lower the risk of complications. Moreover, it is essential to consider the quantity and processing techniques of foods rich in polyphenols to optimize their potential benefits.

### Practical implications

Studies (46) suggest that dietary adjustments can prevent and treat lifestyle-related diseases while promoting overall health. The concept of utilizing complementary medicine and food to enhance human well-being is evident as far back as the Chinese classical texts “Huangdi Neijing” and “Qianjin Prescription.” With the advancement of an aging society, dietary management has gained momentum, and “prevention” and “maintenance” have gradually emerged as central themes in human health. The concept of medicinal food therapy represents the harmonious integration of traditional Chinese medicine’s principles encompassing food therapy, medicinal diet, and health preservation. Non-nutrients found in medicinal and food homologs hold potential as adjunctive measures for preventing and treating chronic diseases, while the significance of a plant-based diet has been underscored in various dietary guidelines (12, 47, 48).

The implementation of dietary therapy is the optimal approach to support the prevention of chronic diseases. Consumption of polyphenols has been scientifically proven to exert favorable effects on health, thereby reducing the risk of cancer, cardiovascular diseases, neurodegenerative disorders, and other degenerative conditions through modulation of inflammatory capacity and improvement in metabolic dysregulation. Moreover, it can effectively impede the onset and progression of chronic ailments (49). The increasing awareness of healthcare among individuals has led to a growing focus on the theory of the homology between medicine and food. Homologs of medicine and food possess diverse characteristics, convenient sampling methods, and high safety standards, thereby offering extensive prospects for application in the field of biomedicine.

### Strengths and limitations

The strength of this study lies in the inclusion of exclusively high-quality randomized controlled trials from four different countries, ensuring reliable results. However, certain limitations should be acknowledged. The search strategy employed in this study is limited to Chinese and English articles, potentially resulting in the omission of significant studies and impacting the overall analysis of results. Furthermore, due to the scarcity of included studies on the outcome indicator CRP and substantial heterogeneity among the included articles, a meta-analysis was not conducted.

## Conclusion

The findings demonstrated that the consumption of polyphenol-rich seed-based foods significantly improved lipid profiles and reduced inflammatory markers among individuals diagnosed with coronary heart disease. The diverse array of foods rich in polyphenols provides a safe and effective complementary approach to preventing chronic diseases associated with diet, thereby promoting the prevention and management of cardiovascular conditions. Future research should investigate different dosages and durations of interventions to further elucidate the specific mechanisms underlying the lipid-lowering and anti-inflammatory effects of seed foods abundant in polyphenols, as well as optimize intervention protocols involving these foods.

## Data Availability

The original contributions presented in the study are included in the article/Supplementary material, further inquiries can be directed to the corresponding author/s.

## Footnotes

^1^ https://www.crd.york.ac.uk/PROSPERO/#recordDetails

^2^ http://phenol-explorer.eu/downloads

## References

[1] Duggan JP Peters AS Trachiotis GD Antevil JL. Epidemiology of coronary artery disease. Surg Clin North Am. (2022) 102:499–516. doi: 10.1016/j.suc.2022.01.007 35671770

[2] Ma LY Wang ZW Fan J Hu SS. Interpretation of report on cardiovascular health and diseases in China 2022. Chin Gen Pract. (2023) 26:3975–94. doi: 10.12114/j.issn.1007-9572.2023.0408

[3] Drakopoulou M Toutouzas K Stathogiannis K Synetos A Trantalis G Tousoulis D. Managing the lipid profile of coronary heart disease patients. Expert Rev Cardiovasc Ther. (2016) 14:1263–71. doi: 10.1080/14779072.2016.1221341 27552726

[4] Li H Sun K Zhao R Hu J Hao Z Wang F . Inflammatory biomarkers of coronary heart disease. Front Biosci (Schol Ed). (2018) 10:185–96. doi: 10.2741/s508, PMID: 28930526

[5] Lan Y Luo FK Yu Y Wang XY Wang PQ Xiong XJ. Coronary heart disease: innovative understanding from traditional Chinese medicine and treatment by classic herbal formulas. China J Chin Mater. 2024, 49:3684–92. doi: 10.19540/j.cnki.cjcmm.20240326.501 39041141

[6] Khan H Kanny O Syed MH Qadura M. Aspirin resistance in vascular disease: a review highlighting the critical need for improved point-of-care testing and personalized therapy. Int J Mol Sci. (2022) 23:1317. doi: 10.3390/ijms231911317, PMID: 36232618 PMC9570127

[7] Khadanga S Beebe-Peat T. Optimal medical therapy for stable ischemic heart disease in 2024: focus on exercise and cardiac rehabilitation. Med Clin North Am. (2024) 108:509–16. doi: 10.1016/j.mcna.2023.11.005, PMID: 38548460

[8] Giustino G Colombo A Camaj A Yasumura K Mehran R Stone GW . Coronary in-stent restenosis: JACC state-of-the-art review. J Am Coll Cardiol. (2022) 80:348–72. doi: 10.1016/j.jacc.2022.05.017 35863852

[9] Wang J Zhang YL Zhong Y Liu ZW Li WZ Xia L . Research advance of anxiety and depression in patient with percutaneous coronary intervention. Chin Gen Gen Pract. (2020) 23:2938–43. doi: 10.12114/j.issn.1007-9572.2019.00.668

[10] Trautwein EA Catapano AL Tokgözoğlu L. 'Diet and lifestyle' in the management of dyslipidaemia and prevention of CVD-understanding the level of knowledge and interest of European atherosclerosis society members. Atheroscler Suppl. (2020) 42:e9–e14. doi: 10.1016/j.atherosclerosissup.2021.01.003, PMID: 33589228

[11] Durazzo A Lucarini M Souto EB Cicala C Caiazzo E Izzo AA . Polyphenols: a concise overview on the chemistry, occurrence, and human health. Phytother Res. (2019) 33:2221–43. doi: 10.1002/ptr.6419, PMID: 31359516

[12] Han SF Feng YQ Gao WQ. Theoretical model of non-nutrient diet therapy for prevention and treatment of chronic diseases. Chin Nurs Res. (2023) 37:565–9. doi: 10.12102/j.issn.1009-6493.2023.04.001

[13] Qorbani M Mahdavi-Gorabi A Khatibi N Ejtahed HS Khazdouz M Djalalinia S . Dietary diversity score and cardio-metabolic risk factors: an updated systematic review and meta-analysis. Eat Weight Disord. (2022) 27:85–100. doi: 10.1007/s40519-020-01090-4 33772731

[14] Li H Zou L Li XY Wu DT Liu HY Li HB . Adzuki bean (*Vigna angularis*): chemical compositions, physicochemical properties, health benefits, and food applications. Compr Rev Food Sci Food Saf. (2022) 21:2335–62. doi: 10.1111/1541-4337.12945, PMID: 35365946

[15] Page MJ McKenzie JE Bossuyt PM Boutron I Hoffmann TC Mulrow CD . The PRISMA 2020 statement: an updated guideline for reporting systematic reviews. J Clin Epidemiol. (2021) 134:178–89. doi: 10.1016/j.jclinepi.2021.03.001, PMID: 33789819

[16] Cumpston M Li T Page MJ Chandler J Welch VA Higgins JP . Updated guidance for trusted systematic reviews: a new edition of the Cochrane handbook for systematic reviews of interventions. Cochrane Database Syst Rev. (2019) 10:ED000142. doi: 10.1002/14651858.ED000142 31643080 PMC10284251

[17] Higgins JPT Thomas J Chandler J Cumpston M Li T Page MJ . *Cochrane handbook for systematic reviews of interventions version 6.4 (updated august 2023). Cochrane* (2023).

[18] Cardozo L Mafra D Silva ACT Barbosa JE da Cruz BO Mesquita CT . Effects of a Brazil nut-enriched diet on oxidative stress and inflammation markers in coronary artery disease patients: a small and preliminary randomised clinical trial. Nutr Bull. (2021) 46:139–48. doi: 10.1111/nbu.12495

[19] Chen CY Holbrook M Duess MA Dohadwala MM Hamburg NM Asztalos BF . Effect of almond consumption on vascular function in patients with coronary artery disease: a randomized, controlled, cross-over trial. Nutr J. (2015) 14:61. doi: 10.1186/s12937-015-0049-5, PMID: 26080804 PMC4469426

[20] Coutinho-Wolino KS da Cruz BO Cardozo L Fernandes IA Mesquita CT Stenvinkel P . Brazil nut supplementation does not affect trimethylamine-n-oxide plasma levels in patients with coronary artery disease. J Food Biochem. (2022) 46:e14201. doi: 10.1111/jfbc.14201, PMID: 35467017

[21] Jamshed H Sultan FA Iqbal R Gilani AH. Dietary almonds increase serum HDL cholesterol in coronary artery disease patients in a randomized controlled trial. J Nutr. (2015) 145:2287–92. doi: 10.3945/jn.114.207944, PMID: 26269239

[22] Khandouzi N Zahedmehr A Firoozi A Nasrollahzadehp J. Effects of flaxseed consumption on plasma lipids, lipoprotein-associated phospholipase a (2) activity and gut microbiota composition in patients with coronary artery disease. Nutr Health. (2022) 2022:1016. doi: 10.1177/02601060221091016 35382631

[23] Khandouzi N Zahedmehr A Mohammadzadeh A Sanati HR Nasrollahzadeh J. Effect of flaxseed consumption on flow-mediated dilation and inflammatory biomarkers in patients with coronary artery disease: a randomized controlled trial. Eur J Clin Nutr. (2019) 73:258–65. doi: 10.1038/s41430-018-0268-x, PMID: 30127374

[24] Saleh-Ghadimi S Kheirouri S Golmohammadi A Moludi J Jafari-Vayghan H Alizadeh M. Effect of flaxseed oil supplementation on anthropometric and metabolic indices in patients with coronary artery disease: a double-blinded randomized controlled trial. J Cardiovasc Thorac Res. (2019) 11:152–60. doi: 10.15171/jcvtr.2019.26, PMID: 31384411 PMC6669420

[25] Li JH Zhang YX Gao F. Research on the development status and countermeasures of "medicine and food homology" food industry. Food and Nutrition in China. (2023) 29:5–11. doi: 10.19870/j.cnki.11-3716/ts.2023.09.002

[26] Yu J . Catalogue of homologous raw materials of medicine and food (edition). Oral Care Ind. (2017) 27:24–8. doi: 10.3969/j.issn.2095-3607.2017.06.007

[27] Hou Y Jiang J-G. Origin and concept of medicine food homology and its application in modern functional foods. Food Funct. (2013) 4:1727–41. doi: 10.1039/c3fo60295h, PMID: 24100549

[28] Song DX Jiang JG. Hypolipidemic components from medicine food homology species used in China: pharmacological and health effects. Arch Med Res. (2017) 48:569–81. doi: 10.1016/j.arcmed.2018.01.004, PMID: 29452699

[29] Lu Q Li R Yang Y Zhang Y Zhao Q Li J. Ingredients with anti-inflammatory effect from medicine food homology plants. Food Chem. (2022) 368:130610. doi: 10.1016/j.foodchem.2021.130610 34419798

[30] Jalali-Khanabadi BA Mozaffari-Khosravi H Parsaeyan N. Effects of almond dietary supplementation on coronary heart disease lipid risk factors and serum lipid oxidation parameters in men with mild hyperlipidemia. J Altern Complement Med. (2010) 16:1279–83. doi: 10.1089/acm.2009.0693, PMID: 21114415

[31] Fatahi S Daneshzad E Lotfi K Azadbakht L. The effects of almond consumption on inflammatory biomarkers in adults: a systematic review and Meta-analysis of randomized clinical trials. Adv Nutr. (2022) 13:1462–75. doi: 10.1093/advances/nmab158 34967837 PMC9526836

[32] Phung OJ Makanji SS White CM Coleman CI. Almonds have a neutral effect on serum lipid profiles: a meta-analysis of randomized trials. J Am Diet Assoc. (2009) 109:865–73. doi: 10.1016/j.jada.2009.02.014, PMID: 19394473

[33] Godos J Vitale M Micek A Ray S Martini D Del Rio D . Dietary polyphenol intake, blood pressure, and hypertension: a systematic review and meta-analysis of observational studies. Antioxidants (Basel). (2019) 8:152. doi: 10.3390/antiox8060152 31159186 PMC6616647

[34] Xu Y Le Sayec M Roberts C Hein S Rodriguez-Mateos A Gibson R. Dietary assessment methods to estimate (poly) phenol intake in epidemiological studies: a systematic review. Adv Nutr. (2021) 12:1781–801. doi: 10.1093/advances/nmab017, PMID: 33684195 PMC8483972

[35] Geto Z Molla MD Challa F Belay Y Getahun T. Mitochondrial dynamic dysfunction as a Main triggering factor for inflammation associated chronic non-communicable diseases. J Inflamm Res. (2020) 13:97–107. doi: 10.2147/JIR.S232009, PMID: 32110085 PMC7034420

[36] Seyedsadjadi N Grant R. The potential benefit of monitoring oxidative stress and inflammation in the prevention of non-communicable diseases (NCDs). Antioxidants (Basel). (2020) 10:10015. doi: 10.3390/antiox10010015 PMC7824370 33375428

[37] Micek A Godos J Cernigliaro A Cincione RI Buscemi S Libra M . Total nut, tree nut, and Peanut consumption and metabolic status in southern Italian adults. Int J Environ Res Public Health. (2021) 18:1847. doi: 10.3390/ijerph18041847, PMID: 33672852 PMC7918537

[38] Micek A Godos J Cernigliaro A Cincione RI Buscemi S Libra M . Polyphenol-rich and alcoholic beverages and metabolic status in adults living in Sicily, southern Italy. Food Secur. (2021) 10:383. doi: 10.3390/foods10020383, PMID: 33572478 PMC7916404

[39] Zhang Q Jia Y Zhang Y Wang Y Li X Tian X . The effects of medicinal and food homologous substances on blood lipid and blood glucose levels and liver function in patients with nonalcoholic fatty liver disease: a systematic review of randomized controlled trials. Lipids Health Dis. (2023) 22:137. doi: 10.1186/s12944-023-01900-5, PMID: 37644446 PMC10464055

[40] Del Gobbo LC Falk MC Feldman R Lewis K Mozaffarian D. Effects of tree nuts on blood lipids, apolipoproteins, and blood pressure: systematic review, meta-analysis, and dose-response of 61 controlled intervention trials. Am J Clin Nutr. (2015) 102:1347–56. doi: 10.3945/ajcn.115.110965, PMID: 26561616 PMC4658458

[41] Tenore GC D'Avino M Caruso D Buonomo G Acampora C Caruso G . Effect of Annurca apple polyphenols on intermittent claudication in patients with peripheral artery disease. Am J Cardiol. (2019) 123:847–53. doi: 10.1016/j.amjcard.2018.11.034, PMID: 30573159

[42] Yan LY Cui W. Discussion on the Blend of Medicinal and Dietary Taboo Culture from the Perspective of “Avoiding Pork after Taking Rhizome of Chinese Goldthread”. Med Philos. (2023) 44:55–9. doi: 10.12014/j.issn.1002-0772.2023.22.13

[43] Auclair S Chironi G Milenkovic D Hollman PC Renard CM Mégnien JL . The regular consumption of a polyphenol-rich apple does not influence endothelial function: a randomised double-blind trial in hypercholesterolemic adults. Eur J Clin Nutr. (2010) 64:1158–65. doi: 10.1038/ejcn.2010.135 20683465

[44] Koutsos A Riccadonna S Ulaszewska MM Franceschi P Trošt K Galvin A . Two apples a day lower serum cholesterol and improve cardiometabolic biomarkers in mildly hypercholesterolemic adults: a randomized, controlled, crossover trial. Am J Clin Nutr. (2020) 111:307–18. doi: 10.1093/ajcn/nqz282, PMID: 31840162 PMC6997084

[45] Zhao GH Zhang RF Su DX Dong LH Liu L Wei ZC . Research progress of whole grain phenols and their antioxidant activities. Chin Inst Food Sci. (2017) 17:183–96. doi: 10.16429/j.1009-7848.2017.08.025

[46] Roy F Boye JI Simpson BK. Bioactive proteins and peptides in pulse crops: pea, chickpea and lentil. Food Res Int. (2010) 43:432–42. doi: 10.1016/j.foodres.2009.09.002

[47] Trautwein EA McKay S. The role of specific components of a plant-based diet in Management of Dyslipidemia and the impact on cardiovascular risk. Nutrients. (2020) 12:2671. doi: 10.3390/nu12092671, PMID: 32883047 PMC7551487

[48] Wu P . Research progress of homologous substances of medicine and food in China. China Fruit Vegetable. (2024) 44:1. doi: 10.3969/j.issn.1008-1038.2024.05.001

[49] Quero J Mármol I Cerrada E Rodríguez-Yoldi MJ. Insight into the potential application of polyphenol-rich dietary intervention in degenerative disease management. Food Funct. (2020) 11:2805–25. doi: 10.1039/D0FO00216J, PMID: 32134090

