# Supplementary material for: Effects of polyphenol-rich seed foods on lipid and inflammatory markers in patients with coronary heart disease: a systematic review
Source: Front Nutr. 2024 Nov 19;11:1493410. doi: 10.3389/fnut.2024.1493410 (PMC11611543; doi:10.3389/fnut.2024.1493410)
Supplement: Supplementary file 1 [file Data_Sheet_1.DOCX]

**Effects of polyphenol-rich seed foods on lipid and inflammatory markers in patients with coronary heart disease: a systematic review**

**The search strategy：**

**Eight databases, including Chinese National Knowledge Infrastructure (CNKI), China Biomedical Literature Database (CBM), Wanfang Database, China Science and Technology Journal Database, PubMed, Cochrane Library, Embase and Web of science, were searched using the combination of MeSH terms and free words. The retrieval period was from the inception of the database to October 9, 2023.**

1. **In Chinese databases, the search was conducted considering the CNKI database as an example of a retrieval strategy:（20）**

**SU=（‘冠心病’+‘****冠状动脉硬化’+‘冠脉硬化’） AND SU=（‘红豆’+‘黑豆’+‘扁豆’+‘栗子’+‘核桃’+‘开心果’+‘蚕豆’+‘榛子’+‘芝麻’+‘葵花’+‘花生’+‘亚麻籽’+‘杏仁’+‘菱角’+‘腰果’+‘鸽豆’+‘豌豆’+‘夏威夷果’+‘大豆’+‘豇豆’+‘鹰嘴豆’+‘绿豆’）**

1. **In Chinese databases, the search was conducted considering the Wanfang database as an example of a retrieval strategy:（148）**

**题名或关键词:(冠心病 or 冠状动脉硬化 or 冠脉硬化) and 题名或关键词:(红豆 or 黑豆 or 扁豆 or 栗子 or 核桃 or 开心果 or** **蚕豆 or 榛子 or 芝麻 or 葵花 or 花生 or 亚麻籽 or 杏仁 or 菱角 or 腰果 or 鸽豆 or 豌豆 or 夏威夷果 or 大豆 or 豇豆 or 鹰嘴豆 or 绿豆)**

1. **In Chinese databases, the search was conducted considering the VIP database as an example of a retrieval strategy:(122)**

**M=（冠心病 OR 冠状动脉硬化** **OR 冠脉硬化) AND M=(红豆 OR 黑豆 OR 扁豆 OR 栗子 OR 核桃 OR 开心果 OR 蚕豆 OR 榛子 OR 芝麻 OR 葵花 OR 花生 OR 亚麻籽 OR 杏仁 OR 菱角 OR 腰果 OR 鸽豆 OR 豌豆 OR 夏威夷果 OR 大豆 OR 豇豆 OR 鹰嘴豆 OR 绿豆)**

1. **In Chinese databases, the search was conducted considering the CBM database as an example of a retrieval strategy:（429）**

**(("红豆"[常用字段:智能] OR "黑豆"[常用字段:智能] OR "扁豆"[常用字段:智能] OR "栗子"[常用字段:智能] OR "核桃"[常用字段:智能]) OR ("开心果"[常用字段:智能]** **OR "蚕豆"[常用字段:智能] OR "榛子"[常用字段:智能] OR "芝麻"[常用字段:智能] OR "葵花"[常用字段:智能] OR "花生"[常用字段:智能] OR "亚麻籽"[常用字段:智能] OR "杏仁"[常用字段:智能] OR "腰果"[常用字段:智能] OR "鸽豆"[常用字段:智能] OR "豌豆"[常用字段:智能] OR "大豆"[常用字段:智能] OR "豇豆"[常用字段:智能] OR "鹰嘴豆"[常用字段:智能] OR "绿豆"[常用字段:智能] OR "夏威夷果"[常用字段:智能] OR "菱角"[常用字段:智能])) AND (****"冠心病"[常用字段:智能] OR "冠状动脉硬化"[常用字段:智能] OR "冠脉硬化"[常用字段:智能])**

1. **For English databases, Pubmed was used as an example:（335）**

**(((("Coronary Disease"[Title/Abstract]) OR ("****coronary heart disease"[Title/Abstract])) OR (CAD[Title/Abstract])) OR ("Coronary Artery Disease"[Mesh])) AND (****((((((((((((((((((((("adzuki bean"[Title/Abstract]) OR ("black soya bean"[Title/Abstract])) OR (Lentils[Title/Abstract])) OR (Chestnut[Title/Abstract])) OR (Walnut[Title/Abstract])) OR (Pistachio[Title/Abstract])) OR ("Broad bean"[Title/Abstract])) OR (Hazelnut[Title/Abstract])) OR (Sesame[Title/Abstract])) OR (Sunflower[Title/Abstract])) OR (Peanut[Title/Abstract])) OR (Flaxseed[Title/Abstract])) OR (Almond[Title/Abstract])) OR ("Brazil nut"[Title/Abstract])) OR ("Cashew nut"[Title/Abstract])) OR ("Pigeon pea"[Title/Abstract])) OR ("pea"[Title/Abstract])) OR ("Macadamia nut"[Title/Abstract])) OR (Soy[Title/Abstract])) OR (Cowpea[Title/Abstract])) OR (Chickpea[Title/Abstract])) OR ("Mung bean"[Title/Abstract]))**

1. **For English databases, Embase was used as an example: (440)**

**('adzuki bean':ab,ti OR 'black soya bean':ab,ti OR lentils:ab,ti OR chestnut:ab,ti OR walnut:ab,ti OR pistachio:ab,ti OR 'broad bean':ab,ti OR hazelnut:ab,ti OR sesame:ab,ti OR sunflower:ab,ti OR peanut:ab,ti OR flaxseed:ab,ti OR almond:ab,ti OR 'brazil nut':ab,ti OR 'cashew nut':ab,ti OR 'pigeon pea':ab,ti OR pea:ab,ti OR 'macadamia nut':ab,ti OR soy:ab,ti OR cowpea:ab,ti OR chickpea:ab,ti OR 'mung bean':ab,ti) AND (****‘CAD’:ab,ti OR** **‘Coronary Disease’:ab,ti OR 'coronary heart disease':ab,ti)**

1. **For English databases, Web of science was used as an example: (50)**

**TI=(adzuki bean OR black soya bean OR Lentils OR Chestnut OR Walnut OR Pistachio OR Broad bean OR Hazelnut OR Sesame OR Sunflower OR Peanut OR Flaxseed OR Almond OR Brazil nut OR Cashew nut OR Pigeon pea OR pea OR Macadamia nut OR Soy OR Cowpea OR Chickpea OR Mung bean）AND TI=(Coronary Disease OR coronary heart disease OR CAD)**

1. **For English databases, The Cochrane library was used as an example:(194)**

**((adzuki bean):ti,ab,kw OR (black soya bean):ti,ab,kw OR (Lentils):ti,ab,kw OR (Chestnut):ti,ab,kw OR (Walnut):ti,ab,kw OR (Broad bean):ti,ab,kw OR (Pistachio):ti,ab,kw OR (Hazelnut):ti,ab,kw OR (Sesame):ti,ab,kw OR (Sunflower):ti,ab,kw OR (Peanut):ti,ab,kw OR (Flaxseed):ti,ab,kw OR (Almond):ti,ab,kw OR (Brazil nut):ti,ab,kw OR (Cashew nut):ti,ab,kw OR (Pigeon pea):ti,ab,kw OR (pea):ti,ab,kw OR (Macadamia nut):ti,ab,kw OR (Soy):ti,ab,kw OR (Cowpea):ti,ab,kw OR (Chickpea):ti,ab,kw OR (Mung bean):ti,ab,kw)AND (****(Coronary Disease):ti,ab,kw OR (coronary heart disease):ti,ab,kw OR (CAD):ti,ab,kw)**
